# Field Test of Excess Pore Water Pressure at Pile–Soil Interface Caused by PHC Pipe Pile Penetration Based on Silicon Piezoresistive Sensor

**DOI:** 10.3390/s20102829

**Published:** 2020-05-16

**Authors:** Yonghong Wang, Xueying Liu, Mingyi Zhang, Suchun Yang, Songkui Sang

**Affiliations:** 1 College of Civil Engineering, Qingdao University of Technology, Qingdao 266033, China; wangyonghong@qut.edu.cn (Y.W.); liuxueying@hnu.edu.cn (X.L.); 15243639294@163.com (S.Y.); 18306426194@163.com (S.S.); 2 Collaborative Innovation Center of Engineering Construction and Safety in Shandong Blue Economic Zone, Qingdao University of Technology, Qingdao 266033, China

**Keywords:** PHC pipe pile, pile jacking, pile–soil interface, excess pore water pressure, field test

## Abstract

Prestressed high-strength concrete (PHC) pipe pile with the static press-in method has been widely used in recent years. The generation and dissipation of excess pore water pressure at the pile–soil interface during pile jacking have an important influence on the pile’s mechanical characteristics and bearing capacity. In addition, this can cause uncontrolled concrete damage. Monitoring the change in excess pore water pressure at the pile–soil interface during pile jacking is a plan that many researchers hope to implement. In this paper, field tests of two full-footjacked piles were carried out in a viscous soil foundation, the laws of generation and dissipation of excess pore water pressure at the pile–soil interface during pile jacking were monitored in real time, and the laws of variation in excess pore water pressure at the pile–soil interface with the burial depth and time were analyzed. As can be seen from the test results, the excess pore water pressure at the pile–soil interface increased to the peak and then began to decline, but the excess pore water pressure after the decline was still relatively large. Test pile S1 decreased from 201.4 to 86.3 kPa, while test pile S2 decreased from 374.1 to 114.3 kPa after pile jacking. The excess pore water pressure at the pile–soil interface rose first at the initial stage of consolidation and dissipated only after the hydraulic gradient between the pile–soil interface and the soil surrounding the pile disappeared. The dissipation degree of excess pore water pressure reached about 75–85%. The excess pore water pressure at the pile–soil interface increased with the increase in buried depth and finally tended to stabilize.

## 1. Introduction

The pile foundation has the advantages of high bearing capacity, strong resistance to non-uniform deformation, and high degree of mechanized construction, so it is widely used in industrial and civil buildings with a self-heavy superstructure, low strength of foundation soil, and high compressibility [1,2,3,4]. With the development of marine economy and high-speed traffic engineering in the 21st century, offshore engineering and high-speed railways and expressways, which can bear complicated loads such as wind, wave, and traffic for a long time, have put forward higher requirements for pile foundations [5,6,7,8]. Prestressed high-strength concrete (PHC) pipe pile has been widely used for its advantages of convenient construction, high bearing capacity, reliable quality, and short molding and curing time [9,10]. Plie jacking produces less noise and vibration, less air pollution, and does not affect the surrounding environment. It is a relatively environmentally friendly construction technology [11,12]. This method can show the pile force in the process of pile jacking, so the PHC pipe pile is widely used in combination with the pile jacking method because of its advantages such as high bearing capacity, strong penetration force, low cost, and environmental protection [13,14,15].

When the pile foundation is jacked into the cohesive soil, the soil around the pile is subjected to extrusion pressure, which can sometimes exceed the self-weight pressure of the overlying soil layer within the range of one pile diameter, and the value of the excess pore water pressure at the pile–soil interface will be larger. At the same time, the influence of pore water on the concrete structure is also very great [16]. At present, a lot of achievements have been made at home and abroad on the excess pore water pressure caused by PHC pipe pile jacking. Cooke and Price [17] pushed the test pile into the overconsolidated London clay and tested the pore water pressure of the soil around the pile after the jacking pile. Roy et al. [18] conducted field tests on the pore water pressure of soil around the pile with static penetration of the jacked pile. Hwang et al. [19] found that the dynamic change in pore pressure was closely related to the penetration process of the pile by embedding sensors in the affected soil layer at a certain distance around the pile to observe the pore water pressure and earth pressure of the full-length pile in the process of pile jacking. Gupta [20] tested the pore water pressure and the horizontal deformation of the surrounding soil on a soft clay foundation. Liu et al. [21] studied the performance of the open pile penetrating into the silty layer based on the indoor model test, and they obtained the accumulation law of the excess pore water pressure in the soil. Burns et al. [22] obtained the variation law of pore water pressure in the permeation process by penetration of jacked piles into fine sand soil, silt soil, and soft soil, and carried out function fitting in combination with the theory of circular hole expansion. Doherty et al. [23], based on the field test, discussed the pore water pressure in the process of pile jacking, and it was found that the end condition has a certain influence on the pore water pressure. Kou et al. [24] studied the law of dissipation of pore water pressure when the jacked pile penetrates into silt-deposited soil based on a field test. Pestana et al. [25] compared and analyzed the dissipation law and influence range of excess pore water pressure based on a large number of field tests, and believed that the detection of excess pore water pressure could effectively predict the property changes of foundation soil. Luo et al. [26] made a comprehensive analysis of a series of pile foundation test data, studied the prevention method of excess pore water pressure in soil, and found that drilling and grooving are effective anti-pressure measures. At present, most domestic and foreign scholars adopt the traditional strain gauge as the monitoring method. Although these studies have made a great contribution to the mechanical characteristics of the pile–soil interface, the traditional test method has too much influence on the test operation, and the structural strain is small and has the disadvantage of zero drift, so the test results are difficult to be applied in practical engineering. The main research content is the change in pore water pressure of soil around the pile caused by the penetration process, and the change in excess pore water pressure at the pile–soil interface is not measured.

The generation of excess pore water pressure at the pile–soil interface affects the lateral friction resistance of the pile during the process of pile jacking. The effective radial stress at the pile–soil interface increases with the dissipation of excess pore water pressure, and the shear strength at the pile–soil interface increases accordingly, which affects the radial stress of the pile body. Therefore, it is urgent to study the changes in excess pore water pressure in the pile–soil interface shear process in cohesive soil. In view of the lack of research on the influence of excess pore water pressure on the pile–soil interface resistance at present, this paper conducted field tests by installing a micro-silicon piezoresistive pore water pressure sensor on the surface of a PHC pipe pile, and measured the change process of excess pore water pressure at the pile–soil interface during pile jacking. It lays the foundation for further study on the influence of excess pore water pressure on the mechanical properties of the pile–soil interface, and proves that the change law of excess pore water pressure has important engineering significance for pile foundation design.

## 2. Silicon Piezoresistive Sensor

**Figure 1:**
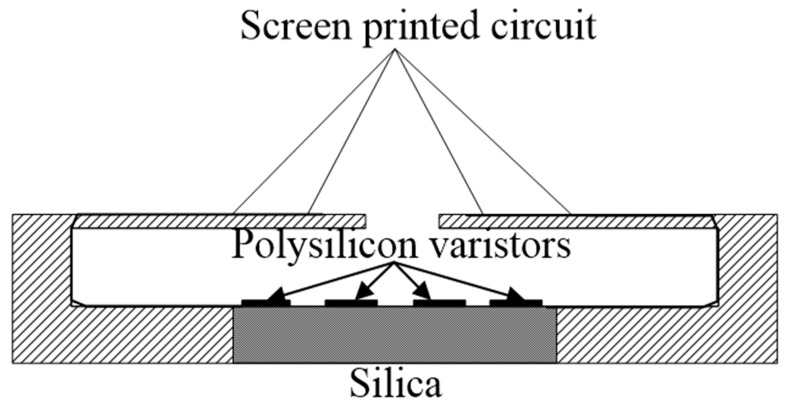
Silicon piezoresistive pressure sensors.

**Figure 2:**
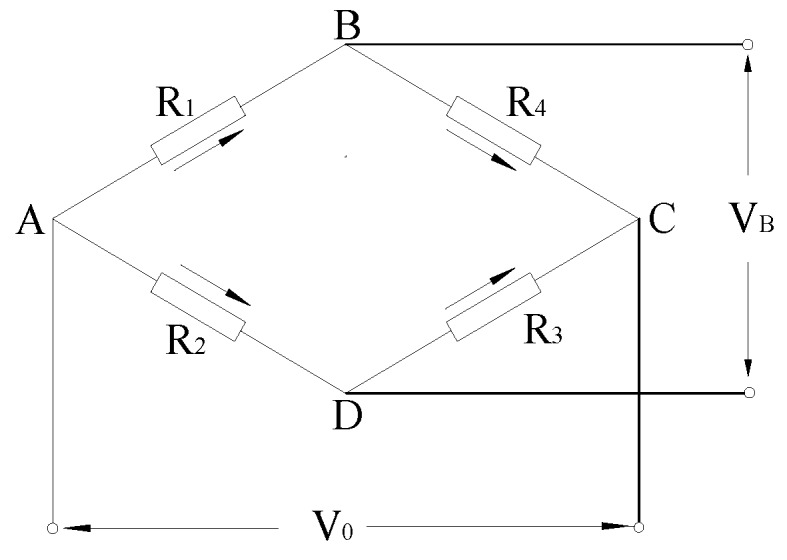
Wheatstone bridge circuit.

With the continuous improvement in accuracy requirements for test results, sensors have been widely used in civil engineering monitoring [27,28,29]. The silicon piezoresistive pressure sensor is a sensitive element with four insulating layers of silicon dioxide pressure-sensitive resistors made of a silicon pressure diaphragm by using the piezoresistive effect with high sensitivity of the polysilicon material. Four varistors of the silicon diaphragm are made to output different voltages through a Wheatstone bridge, and the strain value of the silicon diaphragm is determined according to the voltage value, to obtain the sensitivity coefficient of the sensor. The structure diagram of the silicon piezoresistive pressure sensor is shown in Figure 1, and the Wheatstone bridge circuit is shown in Figure 2 [30].

The output voltage of the Wheatstone bridge is

(1) $$V_{0}=\frac{\left[(R_{1}+\Delta R_{1})(R_{3}+\Delta R_{3})-(R_{2}-\Delta R_{2})(R_{4}-\Delta R_{4})\right]}{\left(R_{1}+R_{2}+\Delta R_{1}-\Delta R_{2})(R_{3}+R_{4}+\Delta R_{3}-\Delta R_{4}\right)}\times V_{B}$$

where $V_{B}$ is the power supply voltage (*V*); $V_{0}$ is the output voltage (*V*); and $R_{1}=R_{3}=R_{2}=R_{4}=R$, where $\Delta R_{i}=R\cdot GF\cdot \varepsilon_{i}$, i=1,2,3,4, and $\varepsilon_{i}$ is the strain value of the ith resistor; thus:

(2) $$V_{0}=\frac{1}{4}GF\cdot \frac{\varepsilon_{1}+\varepsilon_{3}-\varepsilon_{2}-\varepsilon_{4}}{\left[1+\frac{1}{2}(\varepsilon_{1}+\varepsilon_{2}+\varepsilon_{3}+\varepsilon_{4})\right]}V_{B}$$

When the sensor is designed, the four resistance strain values are made to meet the requirements of $\varepsilon_{1}=\varepsilon_{3}=-\varepsilon_{2}=-\varepsilon_{4}=\varepsilon$. The above formula becomes

(3) $$V_{0}=GF\cdot \varepsilon \cdot V_{B}$$

where GF is the strain coefficient, GF=1+2υ+πE, the polysilicon material GF=72.4∼149.6, and the metal material GF=1.5∼2.0. The polysilicon material has a higher sensitivity coefficient than the metal material.

**Figure 3:**
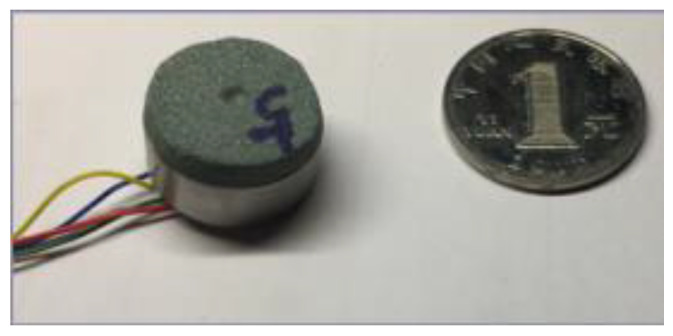
Photos of micro-silicon piezoresistive pressure sensors.

The type of the pore water pressure sensor is a CYY2 dynamic and static pore water pressure sensor. The silicon piezoresistive pressure sensor uses polysilicon as the pressure-sensitive resistor and adopts Micro Electro Mechanical Systems(MEMS) miniaturization technology integration. Permeable stone is placed at the end of the pore water pressure sensor, and the pore water enters through the permeable stone and causes a stress change to the polysilicon pressure-sensitive resistor. A photo of the miniature silicon piezoresistive pressure sensor is shown in Figure 3.

## 3. Engineering Geological Survey of the Test Site

**Figure 4:**
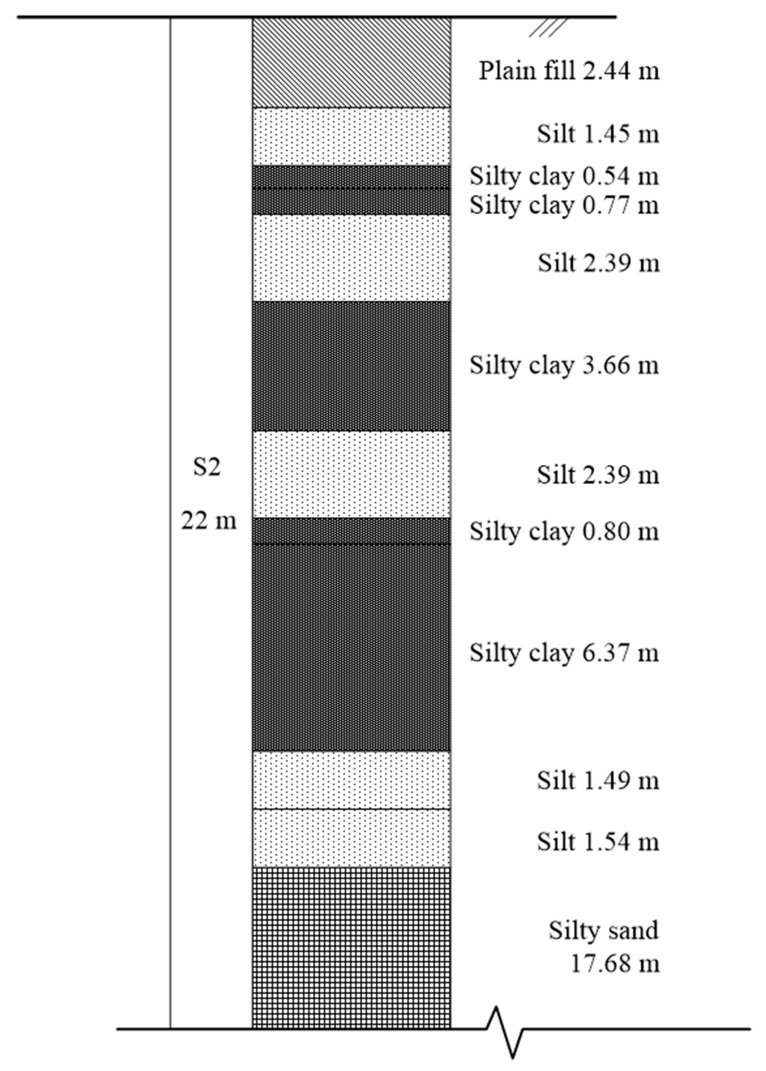
Soil layer distribution.

The construction project site is located in Hekou district, Dongying city, with relatively flat terrain and large local fluctuations. The geomorphologic unit belongs to the quaternary alluvial plain of the Yellow River delta, with a single geomorphologic type, relatively simple stratigraphic structure, relatively uniform horizontal distribution, and vertical layer by layer distribution. According to the exploration, except the plain fill on the surface, the substratum is composed of silt, silty clay, and silty sand in the quaternary alluvium of the Yellow River delta. According to its alluvial genetic types and physical and mechanical indicators, the foundation soil layer within 30 m longitudinally is divided into 12 layers. The distribution of soil layers is shown in Figure 4. The physical and mechanical indicators of each foundation soil layer are shown in Table 1. The underground water level of the site is 0.30–3.00 m.

## 4. Test Pile Conditions and Measuring Point Arrangement

### 4.1. Conditions of Test Pile

The PHC pipe pile of this project is located in a high-rise residential building. According to the proposed building structure form and load situation, combined with the geotechnical engineering site condition and local construction experience, the area residential building adopts the C80-concrete-strength pile body of PHC 400 AB 95-type prestressed concrete pipe pile, where the pile diameter is 400 mm, its thickness is 95 mm, the design of the pile length is 12 and 22 m, and the construction method is the jack method. Two PHC test piles are to be jacked and the pile positions are to be selected as #33 and #169. The designed pile length of #33 is 12 m, the limit value of the designed single-pile bearing capacity is 400 kN, and the designed pile length of #169 is 22 m. The construction is divided into two sections (11 m/section), and the limit value of the designed single-pile bearing capacity is 1500 kN.

In this experiment, the change in excess pore pressure at the pile–soil interface during single-pile jacking was studied. Two test piles, 33# and 169#, were first jacked into the pile before other engineering piles were jacked, and the serial number was S1 and S2 in turn. According to the existing test results [31], the horizontal distance of the soil squeezing effect of jacking piles is generally no more than 15 D (D is the pile diameter). The distance between S1 and S2 test piles is about 20 m, which can avoid the influence of adjacent piles on the test piles during pile jacking.

### 4.2. Arrangement of Measuring Points

**Figure 5:**
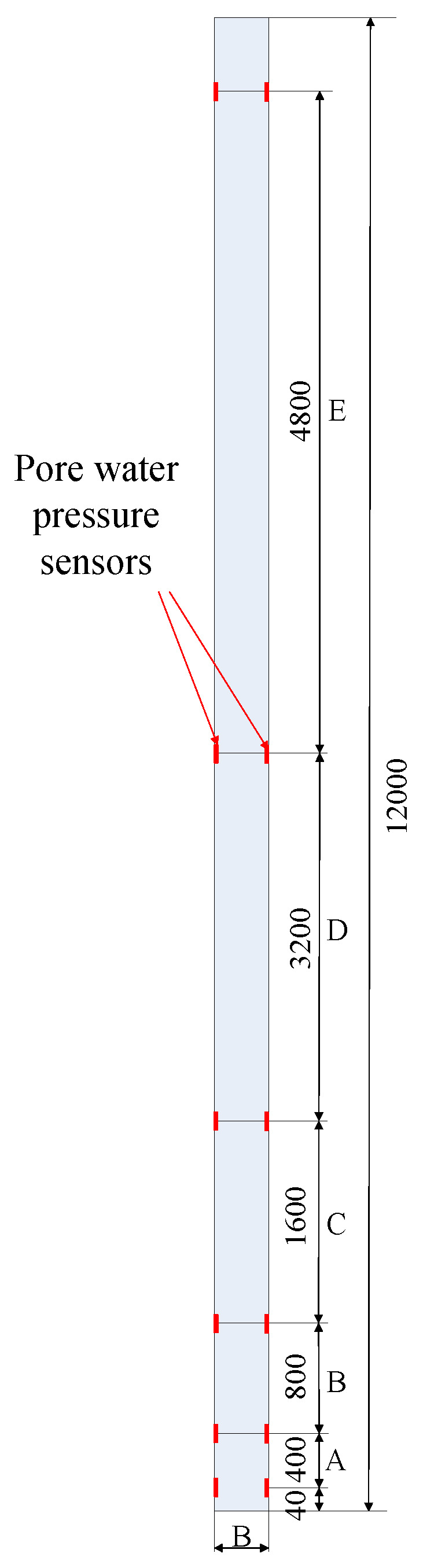
Schematic diagram of pore water pressure sensor along pile of S1 test pile (unit: mm).

In this test, miniature silicon piezoresistive sensors are arranged on the surface of the pile, and pore water pressure sensors are symmetrically installed on the same section on both sides of the pile. In order to avoid the PHC pipe pile end plate, the distance of 40 mm is taken in setting the bottom of the pore water pressure sensors, and the S1 test pile is spaced from the other sensors respectively by D, 2D, 4D, 8D, and 12D (D is the pile diameter), namely 400, 800, 1600, 3200, and 4800 mm, respectively marked as section A, B, C, D, and E. The layout diagram of pore water pressure sensors of test pile S1 along the pile body is shown in Figure 5. The pore water pressure sensors of test pile S2 along the pile body is the same as those of test pile S1.

**Figure 6:**
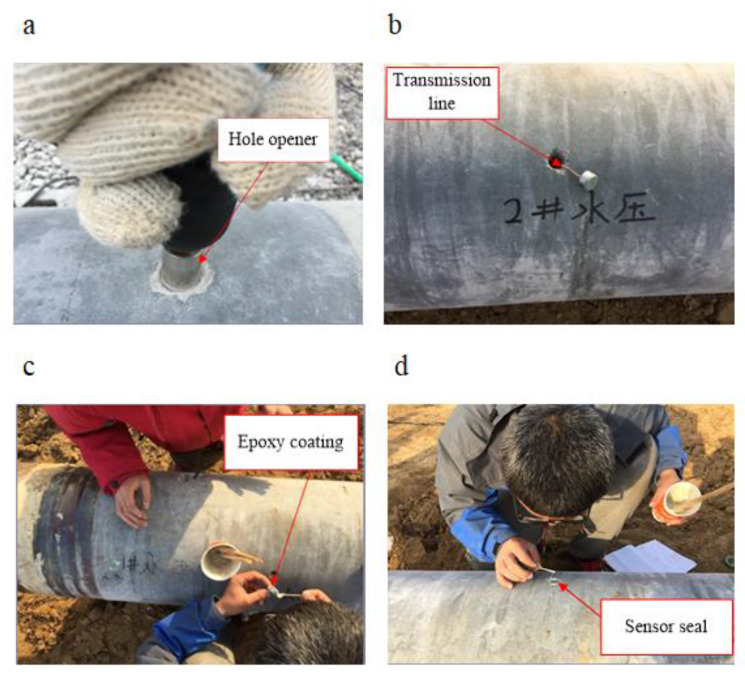
Pore water pressure sensor: (**a**) Hole opener, (**b**) transmission line, (**c**) epoxy coating, (**d**) sensor seal.

According to the testing requirements of the pile jacking process, the pore water pressure sensor at the pile–soil interface must be specially designed to be successfully placed on the surface of the pile body, and the following three conditions must be met: Sensor survival, sensor surface, and pile surface level sensor transmission line from the pile core effectively. Therefore, the key to successfully testing the pore water pressure at the pile–soil interface is the setting method of the pore water pressure sensor. When setting, to make the sensor surface flush with the surface of the pile body, a drill bit with a diameter of 12 mm and a hammer should first be used drill holes in the surface of pile body, and then that with a sensor diameter of 20 mm should be used to punch openings in the hammer-drilled holes, with a hole diameter depth of 20 to 12 mm; the hole size should be in strict accordance with the size of the sensor design. The hole opening by the hole opener is shown in Figure 6a. The pore water pressure sensor is placed in the hole on the surface of the pile, and the sensor transmission line is first introduced into the pile core from the hole before the sensor is placed, as shown in Figure 6b. Then, epoxy resin should be applied to the side of the sensor, as shown in Figure 6c. Finally, the sensor mounted on the surface of the pile was sealed with epoxy resin and cured for 24 h, as shown in Figure 6d. In addition, the surface of the pore water pressure sensor is protected by transparent plastic to prevent the permeable stone from blocking before the pile is jacked. The day before the pile was jacked, the permeable stone of the pore water pressure sensor was filled with water, and the air in the cavity of the pore water pressure sensor was soaked with water for 24 h to discharge.

## 5. Test Results and Analysis

### 5.1. Development Law of Pore Water Pressure at Pile–Soil Interface during Pile Jacking

**Figure 7:**
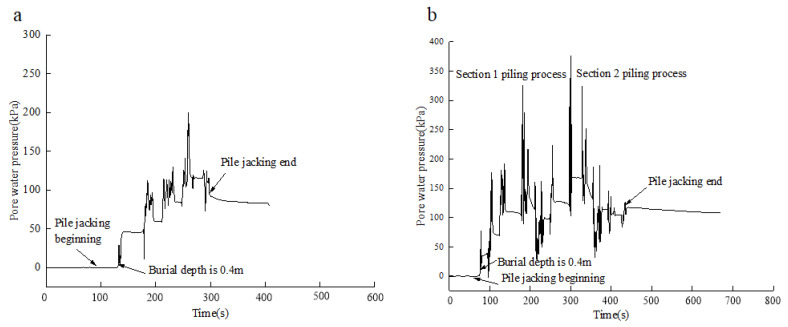
Total pore water pressure of pile–soil interface during pile-jacking (the data came from the sensor test results with h/B = 1 from the pile end): (**a**) Test pile S1 jacking process, (**b**) test pile S2 jacking process.

*h/D* = 1 (*h* is the distance between the pore water pressure sensor and the pile end; *D* is the pore water pressure sensor of test pile diameter) as an example. Figure 7 shows the change law of pore water pressure at the pile–soil interface during pile jacking. The S1 test pile jacking time is 09:52:57–09:55:20 on November 27, 2017, and the S2 test pile jacking time is 10:54:00–10:59:48 on November 27, 2017.

Figure 7 shows the change law of the total pore water pressure at the S1 and S2 test pile pile–soil interface during the process of pile jacking. As can be seen from the figure, the pore water pressure at the pile–soil interface increases with the increase in burial depth overall. Further analysis shows that:

(1) At the initial stage of pile jacking, the excess pore water pressure at the pile–soil interface increased sharply, indicating that the soil was extruded and expanded when shearing occurred at the pile–soil interface, which resulted in the increase in excess pore water pressure at the pile–soil interface with the burial depth. Through field observation, Tang et al. [32,33] also found the same change law.
(2) During pile jacking, the excess pore water pressure at the pile–soil interface increased to the peak and then began to decline, but the excess pore water pressure was still large after the decline. At the end of the S1 pile jacking, the excess pore water pressure from the pile end position *h/B* = 1 decreased from 201.4 to 86.3 kPa. After the end of pile jacking in the upper section of test pile S2, the excess pore water pressure from the pile end position *h/B* = 1 decreased from the maximum 324.6 to 39.2 kPa. It can be seen that the excess pore water pressure dissipates rapidly in the interval of pile jacking, and, after the end of pile jacking in the under section of test pile S2, the pressure drops from 374.1 to 114.3 kPa. The pore water pressure sensors measured at different positions *h/B* from the pile end have the same variation law as above.
(3) After the completion of pile jacking, there is a hydraulic gradient between the pile–soil interface and the soil around the pile, and the pore water pressure in the soil around the pile will transfer to the pile–soil interface under the action of seepage. Therefore, the pore water pressure at the pile–soil interface will be stable within 24 h in the early stage of consolidation and will rise before the disappearance of the hydraulic gradient. After the hydraulic gradient disappeared completely, the pore water pressure began to dissipate, and the pore water pressure curve showed a downward trend.

### 5.2. Dissipation Law of Pore Water Pressure at Pile–Soil Interface during Pile Jacking

**Figure 8:**
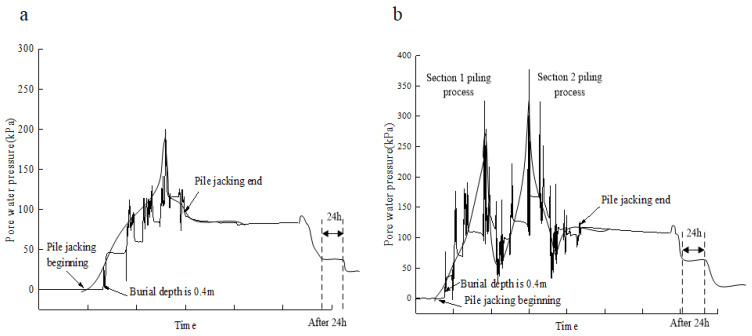
Curves of excess pore water pressure of pile–soil interface during pile jacking (the data came from the sensor test results with h/B = 1 from the pile end): (**a**) Test pile S1 pile jacking process, (**b**) test pile S2 pile jacking process.

As the test site is a viscous soil layer above 20 m, the study is conducted on the basis of the dissipation law of excess pore water pressure at the pile–soil interface in the viscous soil, and the pore water pressure sensor *h/B* = 1 (*h* is the distance between the pore water pressure sensor and the pile end; *B* is the pile diameter of the test pile) from the pile end is still taken as an example. Figure 8 shows the relationship curve of the generation and dissipation of excess pore water pressure at the pile–soil interface with time during pile jacking. The dissipation results of pore water pressure within 48 h after the completion of pile jacking are shown in Table 2.

As can be seen from Figure 8:

(1) Unlike the dissipation law of pore water pressure in the soil around the pile, the dissipation law of excess pore water pressure at the pile–soil interface after the pile jacking end first goes up and then down. The reason is that the pressure difference between the pile–soil interface and the soil around the pile occurs after the construction, that is, a hydraulic gradient exists between the pile–soil interface and the soil around the pile, and the pore water pressure in the soil around the pile will transfer to the pile–soil interface under the action of seepage. Therefore, the excess pore water pressure at the pile–soil interface rises first at the initial stage of consolidation, and the pressure difference between the pile–soil interface and the soil surrounding the pile does not exist until the hydraulic gradient disappears.
(2) The excess pore water pressure of the test pile S1 and S2 pile–soil interface measured within 24 h after becoming basically stable was 41 and about 65 kPa, respectively, and at this time, the excess pore water pressure dissipation degree had reached 75%–85%, and the dispersal rate is obviously more than the soil consolidation theory calculation speed, namely on the field test of the pile–soil interface excess pore water pressure dissipation rate and soil consolidation theory calculation speed. The permeability coefficient of the viscous soil layer is about 1.0 × 10^–7^ cm/s and the coefficient of consolidation *C*_v_ is about 6.0 × 10^–3^ cm^2^/s. The field-measured pore water pressure dissipation rate is obviously faster than the calculation rate based on the consolidation theory around the pile. Some scholars have made corresponding studies [34]. It is generally believed that the hydraulic splitting effect occurs in the soil around the pile due to the excessive pore water pressure generated in the process of continuous pile jacking, resulting in a large number of fractures. As a result, the consolidation speed of soil around the pile was accelerated at the beginning of the end of pile jacking. With time, cracks were gradually bridged, the permeability of soil around the pile was reduced, and the consolidation speed gradually slowed down. Different pore water pressure sensors from the pile to the pile end *h/B* were tested, which had the same variation law as above [35].

### 5.3. Change Law of Excess Pore Water Pressure at the Pile–Soil Interface with the Burial Depth

**Figure 9:**
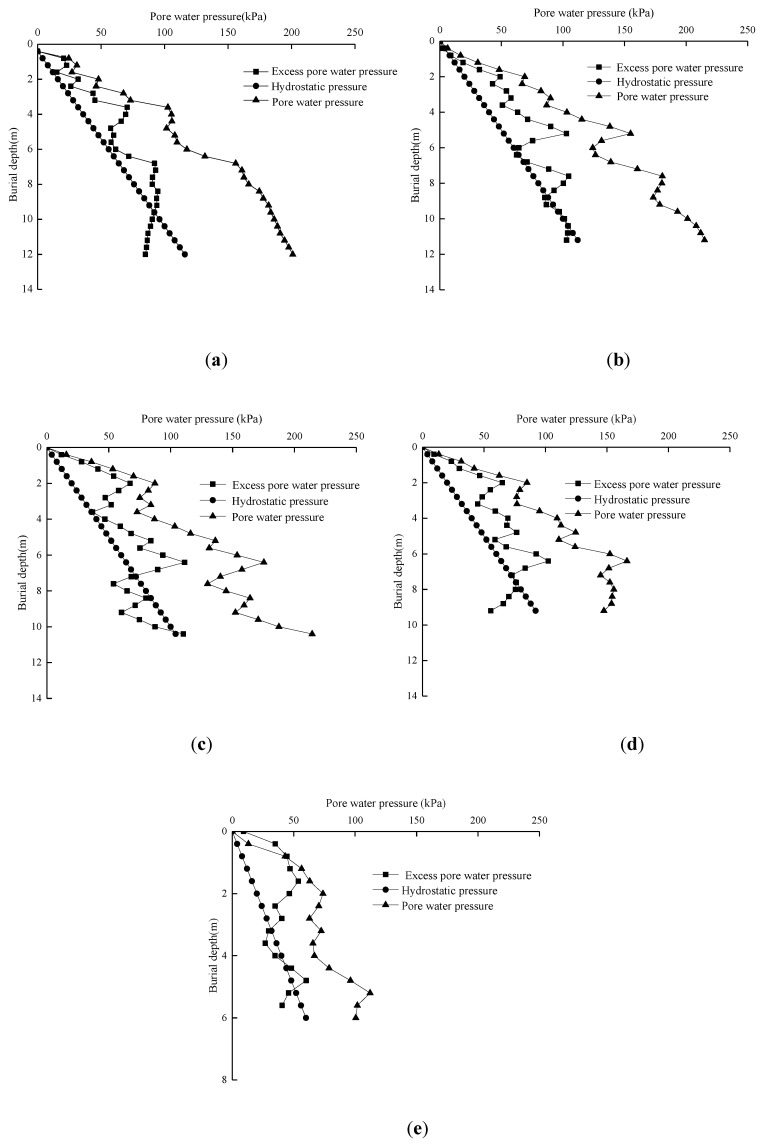
Variation curves of excess pore water pressure of pile–soil interface with depths during pile-jacking: (**a**) h/B = 1, (**b**) h/B = 3, (**c**) h/B = 7, (**d**) h/B = 15, (**e**) h/B = 27.

Taking test pile S1 as an example, Figure 9 shows the variation law of the excess pore water pressure at the pile–soil interface with the burial depth, and the excess pore water pressure in the figure is the measured value after the stability of the pile jacking process. As can be seen from Figure 9a, when the burial depth is less than 4.0 m, the excess pore water pressure at the pile–soil interface increases linearly during the pile jacking process. When the buried depth exceeds 4.0 m, the excess pore water pressure at the pile–soil interface tends to decrease. After the burial depth exceeds 6.0 m, the excess pore water pressure at the pile–soil interface gradually increases to 100 kPa, and the excess pore water pressure at the pile–soil interface is very close to the change trend of the pile side average lateral resistance. The excess pore water pressure at this depth is less affected by the depth. This indicates that the excess pore water pressure has an impact on the change in shear strength at the pile–soil interface during the pile jacking process. The change in shear strength at the pile–soil interface during the pile jacking process can reflect the change trend of excess pore water pressure, which is consistent with the conclusion that the existing excess pore water pressure has an impact on the shear strength at the pile–soil interface [36]. When test pile S1 was buried for 12 m, the total pore water pressure at the pile–soil interface measured by the pore water pressure sensor *h/B* = 1 from the pile end was about 201.4 kPa. At the same time, the change law of total pore water pressure at the pile–soil interface with the burial depth is given in the figure. It can be seen from the figure that the total pore water pressure at the pile–soil interface is approximately linear with the burial depth [37].

## 6. Conclusions

By installing a micro-pore water pressure sensor on the surface of the pile body, conducting field tests to test the jacking pile, obtaining the pore water pressure at the pile–soil interface, and studying the law of generation and dissipation of excess pore water pressure at the pile–soil interface, conclusions can be drawn as follows:

(1) At the initial stage of pile jacking, the excess pore water pressure at the pile–soil interface increased sharply, and the excess pore water pressure increased with the increase in burial depth. At the end of pile jacking, the excess pore water pressure at the pile–soil interface dissipated rapidly, and the excess pore water pressure of test pile S1 decreased from 201.4 to 86.3 kPa, while the excess pore water pressure of test pile S2 decreased from 324.6 to 39.2 kPa.
(2) After the end of pile jacking, the dissipation law of excess pore water pressure at the pile–soil interface is that it rises first and then falls. Due to the existence of a hydraulic gradient, the pore water pressure in the soil around the pile will transfer to the pile–soil interface under the action of seepage. The excess pore water pressure at the pile–soil interface will first rise at the initial stage of consolidation and then dissipate when the hydraulic gradient disappears at the pile–soil interface. With the extension of time, the fracture of the pile–soil interface under the action of hydraulic fracturing increases the dissipation rate of the pile–soil interface.
(3) The excess pore water pressure at the pile–soil interface has an influence on the shear strength change at the pile–soil interface during the process of pile jacking, and the shear strength change at the pile–soil interface during the process of pile jacking can reflect the change trend of excess pore water pressure. Research on the generation and dissipation of excess pore water pressure at the pile–soil interface in the process of pile jacking plays an important role in the pile’s mechanical characteristics and bearing capacity and has important engineering application value.
(4) The dissipation trend of pore water pressure at different positions of the pile body is consistent. After a period of steady state, the pore water pressure dissipates gradually with the increase in time. With the increase in the sensor position, the pore water pressure decreases and the dissipation rate of pore water decreases.

## Figures and Tables

**Table 1:** Physical and mechanical properties of soil layer.

Soil Type	Soil Thickness (m)	Severe *r*/(kN/m^2^)	Moisture Content *w*(%)	Void Ratio *e*	Liquid Limit *w*_L_(%)	Plastic Limit *w*_P_(%)	Cohesion *C*(kPa)	Internal Friction Angle *φ*(°)	Compresson Modulus *E_s_*(MPa)	Characteristic Value of Bearing Capacity *f*_ak_(kPa)
Plain fill	2.44									
Silt	1.45	18.6	27.7	0.792	28.5	19.6	10.0	18.9	6.0	95
Silty clay	0.54	18.4	31.1	0.881	32.5	20.0	16.9	7.3	3.0	80
Silty clay	0.77	18.4	30.5	0.873	32.1	19.7	18.5	10.0	3.0	80
Silt	2.39	18.8	28.0	0.796	28.9	20.0	9.5	19.9	6.5	100
Silty clay	3.66	18.3	31.5	0.892	32.3	19.8	18.8	7.2	3.0	80
Silt	2.39	18.8	28.0	0.793	28.9	19.9	10.0	20.3	7.0	120
Silty clay	0.8	18.5	30.8	0.875	32.6	19.8	17.9	9.9	3.5	85
Silty clay	6.37	18.5	30.8	0.862	32.6	19.9	18.6	10.7	4.0	90
Silt	1.49	18.8	27.7	0.789	28.6	19.7	10.7	20.3	7.0	120
Silt	1.54	19.0	27.0	0.754	28.7	20.2	11.7	22.0	7.5	160
Silty sand	17.68	19.3	24.0	0.692			5.0	34.0	10.3	220

**Table 2:** Dissipation value of pore water pressure within 48 h after pile jacking (unit: kPa).

	Sensor Position	h/B = 1	h/B = 3	h/B = 7	h/B = 15	h/B = 27
Time						
12 h		105	96	88	45	28
24 h		98	86	70	38	19
36 h		92	75	65	33	15
48 h		85	70	61	28	12
